# Neurofibromatosis Type 1 Has a Wide Spectrum of Growth Hormone Excess

**DOI:** 10.3390/jcm11082168

**Published:** 2022-04-13

**Authors:** Fady Hannah-Shmouni, Giampaolo Trivellin, Pablo Beckers, Lefkothea P. Karaviti, Maya Lodish, Christina Tatsi, Adekunle M. Adesina, Fotini Adamidou, Gesthimani Mintziori, Jami L. Josefson, Martha Quezado, Constantine A. Stratakis

**Affiliations:** 1 Section on Endocrinology & Genetics (SEGEN), *Eunice Kennedy Shriver* National Institute of Child Health and Human Development (NICHD), National Institutes of Health, Bethesda, MD 20892, USA; fady.hannah-shmouni@nih.gov (F.H.-S.); giampaolo.trivellin@humanitasresearch.it (G.T.); pablo.beckers@chuliege.be (P.B.); christina.tatsi3@nih.gov (C.T.); 2 Laboratory of Cellular and Molecular Endocrinology, Humanitas Research Hospital—IRCCS, 20089 Rozzano, Italy; 3 Laboratory of Molecular Biology, Department of Human Genetics, University Hospital of Liege, 4000 Liege, Belgium; 4 Department of Pediatrics, Section of Diabetes and Endocrinology, Texas Children’s Hospital, Baylor College of Medicine, Houston, TX 77030, USA; karaviti@bcm.edu; 5 Department of Pediatrics, University of California, San Francisco, CA 94143, USA; maya.lodish@ucsf.edu; 6 Immunology and Pediatrics-Hematology/Oncology, Neuropathology and Molecular Neuropathology, Texas Children’s Hospital, Baylor College of Medicine, Houston, TX 77030, USA; amadesin@texaschildrens.org; 7 Department of Endocrinology, Hippokration General Hospital of Thessaloniki, 54642 Thessaloniki, Greece; fotini1@hol.gr (F.A.); gefsi@auth.gr (G.M.); 8 Division of Endocrinology, Ann and Robert H. Lurie Children’s Hospital of Chicago, Northwestern University Feinberg School of Medicine, Chicago, IL 60611, USA; j-josefson@northwestern.edu; 9 Laboratory of Pathology, National Cancer Institute (NCI), NIH, Bethesda, MD 20892, USA; quezadom@mail.nih.gov; 10 Human Genetics & Precision Medicine, Institute of Molecular Biology and Biotechnology (IMBB), Foundation for Research & Technology Hellas, 70013 Heraklion, Greece; 11 Experimental-Research Center ELPEN, 19009 Athens, Greece

**Keywords:** acromegaly, gigantism, GH excess, neurofibromatosis type 1, overgrowth, optic pathway glioma, X-LAG, GPR101, pituitary tumor

## Abstract

Overgrowth due to growth hormone (GH) excess affects approximately 10% of patients with neurofibromatosis type 1 (NF1) and optic pathway glioma (OPG). Our aim is to describe the clinical, biochemical, pathological, and genetic features of GH excess in a retrospective case series of 10 children and adults with NF1 referred to a tertiary care clinical research center. Six children (median age = 4 years, range of 3–5 years), one 14-year-old adolescent, and three adults (median age = 42 years, range of 29–52 years) were diagnosed with NF1 and GH excess. GH excess was confirmed by the failure to suppress GH (<1 ng/mL) on oral glucose tolerance test (OGTT, *n* = 9) and frequent overnight sampling of GH levels (*n* = 6). Genetic testing was ascertained through targeted or whole-exome sequencing (*n* = 9). Five patients (all children) had an OPG without any pituitary abnormality, three patients (one adolescent and two adults) had a pituitary lesion (two tumors, one suggestive hyperplasia) without an OPG, and two patients (one child and one adult) had a pituitary lesion (a pituitary tumor and suggestive hyperplasia, respectively) with a concomitant OPG. The serial overnight sampling of GH levels in six patients revealed abnormal overnight GH profiling. Two adult patients had a voluminous pituitary gland on pituitary imaging. One pituitary tumor from an adolescent patient who harbored a germline heterozygous p.Gln514Pro *NF1* variant stained positive for GH and prolactin. One child who harbored a heterozygous truncating variant in exon 46 of *NF1* had an OPG that, when compared to normal optic nerves, stained strongly for GPR101, an orphan G protein-coupled receptor causing GH excess in X-linked acrogigantism. We describe a series of patients with GH excess and NF1. Our findings show the variability in patterns of serial overnight GH secretion, somatotroph tumor or hyperplasia in some cases of NF1 and GH excess. Further studies are required to ascertain the link between NF1, GH excess and GPR101, which may aid in the characterization of the molecular underpinning of GH excess in NF1.

## 1. Introduction

Neurofibromatosis type 1 (NF1, MIM: 162,200) is a progressive autosomal dominant condition characterized by an increased risk of benign and malignant tumor formation with phenotypic variability [1,2]. The estimated incidence and prevalence of pediatric NF1 is 30–38 cases per 100,000 live births and 3.0 per 10,000 population (95% confidence interval, 2.3–4.0), respectively [3]. NF1 commonly arises from pathogenic variants or deletions in *NF1* (17q11.2) that codes for neurofibromin. A deficiency of neurofibromin leads to the hyperactivation of the proto-oncogene *RAS* and its downstream effectors [2,4]. The phenotype is broad and includes café-au-lait spots, neurofibromas, freckling in the inguinal and axillary regions, ocular Lisch nodules, and predisposition to multiple tumors (such as optic pathway gliomas, OPGs) and endocrinopathies [1,2].

NF1 has been known to be associated with overgrowth since the mid 20th century [5], while only a few cases of NF1 with overgrowth due to growth hormone (GH) excess have been reported in the medical literature [5,6,7,8,9,10,11,12,13,14,15,16]. In the largest study to date, Cambiaso et al. found that 10% of children with NF1 and OPG have GH excess with or without central precocious puberty [14]. Interestingly, all of their affected children had a tumor consistent with OPG [14], usually identified on magnetic resonance imaging (MRI) scans as contrast enhancing masses [17], and without an obvious pituitary lesion or hyperplasia. Only a few documented cases of NF1 and pituitary tumors with GH excess have been described [7,8,9,10,13,18,19,20], with no known co-existing hyperplasia, or loss of heterozygosity to *NF1*. This was further supported by a study that found no pathogenic variants in *NF1* in non-NF1 related nonfunctioning (*n* = 36) and somatotroph (*n* = 20) tumors, suggesting that the inactivation of neurofibromin may not have a primary role in the formation of pituitary tumors [21]. Although the mechanism underlying GH excess in NF1 is unknown, it has been postulated that the loss of somatostatinergic inhibition from infiltrating OPGs, particularly those involving the hypothalamic and pituitary regions, leads to a dysregulated GH secretion pattern [5,22]. Others have proposed the presence of overexpressed growth hormone releasing hormone (GHRH) in OPGs [23], although staining for GHRH in some cases was negative [5,11,12]. GH excess is generally a rare disease in children and adults, but it appears to affect patients with NF1 at higher rates, and across all ages, potentially increasing their oncological risk or growth of an existing tumor [15,24]. Importantly, GH excess is not recognized as a frequent clinical entity in NF1, and, hence, it is often omitted from NF1-related guidelines [2,25].

Our group has shown that Xq26.3 microduplications that involve the *GPR101* gene and result in its overexpression in pituitary tumors cause X-linked acrogigantism (X-LAG; MIM: 300,942) [26]. This disorder is characterized by early childhood onset gigantism with mixed GH and prolactin-secreting pituitary tumor or hyperplasia before two years of age [27]. *GPR101* (MIM: 300,393) encodes for an orphan G protein-coupled receptor (GPCR) important for brain and pituitary function [28].

Excessive somatic growth in a child with NF1 and OPG has been observed as early as 6 months of life [29], which coincides with the timeline of excessive growth in X-LAG [26]. However, no studies have evaluated the link between NF1 and GPR101. Thus, we seek to report on the clinical, biochemical, pathological, and genetic features of our mixed cohort of NF1 and GH excess, and to investigate the potential role of GPR101 in one case. To our knowledge, this is the most comprehensively examined retrospective case series of pediatric and adult patients presenting with this rare clinical entity.

## 2. Materials and Methods

### 2.1. Patients

Six pediatric, one adolescent, and three adult patients with NF1 were referred to the National Institutes of Health (NIH) over the past two decades for the evaluation of accelerated linear growth, gigantism, or acromegaly, respectively (Table 1 and Table 2). All patients fulfilled the clinical diagnostic criteria for NF1, as reported previously [1,30]. The *Eunice Kennedy Shriver* National Institute of Child Health and Human Development (NICHD) institutional review board approved the study under a clinical protocol that studies the genetics of pituitary tumors and related disorders (protocol 97-CH-0076). Adult patients and legal guardians of pediatric patients gave written and informed consent for clinical and genetic investigations; assent was acquired by pediatric patients when appropriate.

### 2.2. Endocrine, Radiographic and Genetic Investigations

**Figure 1:**
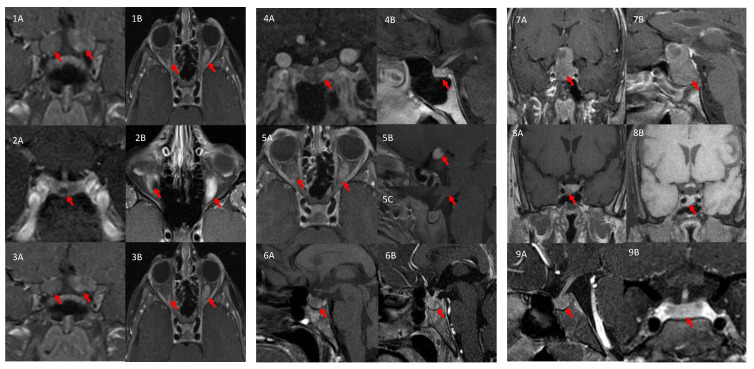
T1 (A) and T2 (B weighted MRI findings in our cohort. 1(A,B) (patient 1): bilateral low grade OPG. 2(A,B) (patient 2): pituitary microadenoma (2(A) arrow) with bilateral low grade OPG (2(B) arrow). 3(A,B) (patient 3): bilateral low grade OPG (arrows). 4(A,B) (patient 5): voluminous pituitary gland (arrows). 5(A–C) (patient 6): bilateral, low grade OPG (5(A), arrows) with a hypothalamic lesion (5(B) and 5(C)), arrow), most likely a glioma. 6(A,B) (patient 7): voluminous pituitary gland (6(A), arrow) with a Rathke’s cyst (6(B), arrow). 7(A,B) (patient 8): large tumor with invasion of the cavernous sinus, and a suprasellar expansion, near the chiasma. 8(A,B) (patient 9): macroadenoma invading into the cavernous sinus. 9(A,B) (patient 10): heterogenous enhancement and voluminous pituitary gland.

GH excess was suspected when linear growth acceleration (crossing >2 major height percentiles) (pediatric cohort) or acromegalic features (adults) were observed in combination with clinical, genetic, and biochemical characteristics. The biochemical evidence of GH excess was demonstrated by elevated IGF-1 levels for age (adjusted for sex and puberty), failure to suppress GH (<1.0 ng/mL) on oral glucose tolerance test (1.75 gm/kg to max of 75 gm of dextrose on the OGTT, *n* = 9), and frequent overnight sampling of GH levels (*n* = 6). GH and prolactin sampling began at 21.00 h, measured every 20 min until 08.00 h next day, and sleep was not interrupted by the blood withdrawal. Night GH secretory profiles were compared for mean GH, pulse amplitude and pulse frequency as detailed elsewhere [31,32]. We used normative data for serial GH sampling that are available from previous studies [33,34,35]. All patients underwent a dedicated MRI of the pituitary gland, brain, and optic tract (Figure 1).

Nine patients underwent genetic testing for *NF1* from peripheral blood DNA, which was performed at the Birmingham Medical Genomics Laboratory of the University of Alabama (UAB: www.uab.edu/medicine/genetics/medical-genomics-laboratory). Chromosomal microarray analysis was performed at either Quest Diagnostics Incorporated Nichols Institute (www.questdiagnostics.com/home/physicians/testing-services/by-test-name/clarisure/cma) or GeneDx (www.genedx.com/test-catalog/molecular-cytogenetics/microarray-analysis). *NF1* gene sequencing as well as the deletion/duplication analysis of the entire coding region plus the alternatively spliced exons 9br, 23a and 48a (60 exons total) were performed from blood lymphocytes using Next-Generation Sequencing version 101 at UAB. This was followed by Sanger confirmatory sequencing and Copy Number (CN) analysis by Multiplex Ligation-Dependent Probe Amplification (MLPA). The limits of uncertainty for the exact location of the breakpoints are defined according to the ligation sites of the MLPA probes flanking the 5′ and 3′ ends of the deletion/duplication. Sanger sequencing and CNV analysis for *GPR101* were performed as previously described [26]. The loss of heterozygosity (LOH) for *NF1* was investigated in DNA extracted from the pituitary tumor of patient 8 by Sanger sequencing, using primers flanking the variant identified by whole-exome sequencing. All variants have been annotated according to Human Genome Variation Society (HGVS) recommendations (www.hgvs.org/mutnomen, accessed on 20 February 2022) and using the NM_000267.3 as reference sequence. Exon naming was assigned by systematic numbering. In silico predictions were performed with Alamut version 2.9.0 (Interactive Biosoftware, Rouen, France). Allele frequencies in the general population were retrieved from the gnomAD database (https://gnomad.broadinstitute.org/, accessed on 20 February 2022).

### 2.3. Immunohistochemical Analysis

Two pituitary tumors, one from a child and one from an adult patient, one OPG from a child, and two tumors resected from one adult—a gastrointestinal stromal tumor (GIST) and a pancreatic neuroendocrine tumor (pNET)—were examined. Formalin-fixed paraffin-embedded samples were preserved and sectioned using standard techniques. All paraffin-embedded slides were submitted to de-paraffinization (Histo-Clear (National Diagnostics HS-200)), rehydration with decreasing ethanol concentrations and antigen retrieval for 30 min in citrate buffer solution (Vector H3300, pH 6.0). The slides were incubated with 10% normal donkey serum for 1 h for blocking of nonspecific binding, and afterward incubated overnight at 4 °C with the following primary antibodies: rabbit anti-GPR101 (dilution 1:500; SAB4503289, Sigma-Aldrich, St. Louis, MO, USA) [28], goat anti-GH (dilution 1:100, sc-10364, Santa Cruz Biotechnology, Santa Cruz, CA, USA), rabbit anti-NF1 (dilution 1:200, HPA045502, Sigma-Aldrich, St. Louis, MO, USA). All slides were incubated for 1 h with 1:1000 Biotin-SP AffiniPure Goat Anti-rabbit IgG (111-065-144, Jackson ImmunoResearch Laboratories, West Grove, PA, USA) and for 30 min with 1:500 Peroxidase Streptavidin (016-030-084, Jackson ImmunoResearch Laboratories, West Grove, PA, USA). Samples were developed by incubation for 1 min with ImmPACT DAB peroxidase (HRP) substrate (Vector SK-4105) and counterstained with Gill’s hematoxylin I (HXGHE1PT, American MasterTech Scientific, McKinney, TX, USA) and Dako Bluing Buffer (CS70230-2, Agilent Technologies, Santa Clara, CA, USA). As negative control, a specimen’s section was incubated under identical conditions without primary antibody. Images were acquired using a Leica DMRX optical microscope, attached to an Olympus DP72 camera, and processed with the CellSens Dimension 1.6 software (Olympus, Hamburg, Germany). Staining was evaluated in 3 high-power fields and samples scored as strongly positive (3+), moderately positive (2+), weakly positive (1+) or negative (0). Staining pattern (membranous, cytoplasmic or nuclear) was also recorded.

## 3. Results

### 3.1. Clinical Studies

#### 3.1.1. Patient 1

A 4-year 8-month-old Caucasian female with NF1 diagnosed in the neonatal period was presented for evaluation of accelerated linear growth. At age 3, she had crossed from the 50th up into the 97th percentile for height. Her growth velocity was ~11 cm/year. She had no evidence of precocious puberty on physical examination. Biochemical evaluation revealed IGF-1 of 540 ng/mL (Z-Score for Tanner 1: 9), and GH 5.1 ng/mL. GH excess was confirmed through overnight GH sampling only. MRI of the pituitary showed bilateral OPGs, with an unremarkable pituitary gland and hypothalamus.

#### 3.1.2. Patient 2

A 5-year-old Caucasian male with NF1 first diagnosed at age 2 was presented for the evaluation of persistent accelerated linear growth since the age of 3. His growth velocity was ~9 cm/year. He had no evidence of precocious puberty on physical examination. Biochemical screening revealed IGF-1 of 659 ng/mL (Z-Score for Tanner 1: 15.3), and GH 13.6 ng/mL. GH excess was confirmed by failure to suppress GH on OGTT (GH nadir = 4.2 ng/mL), and overnight GH sampling. Pituitary MRI showed optic and hypothalamic lesions, likely representing gliomas, and a 4 mm right pituitary tumor. He underwent a transsphenoidal resection of the pituitary lesion. The histopathologic evaluation of the resected tumor for GH was negative.

#### 3.1.3. Patient 3

A 4-year-old South Asian male with NF1 first diagnosed at age 1 was presented for the evaluation of accelerated linear growth. His height had increased from the 50th up to the 95th percentile over the previous year. His growth velocity was on average ~7 cm/year. He had no evidence of precocious puberty on physical examination. At 4 years, he underwent biochemical screening showing IGF-1 of 233 ng/mL (Z-Score for Tanner 1: 3.8). GH excess was confirmed by failure to suppress GH on OGTT (GH nadir = 5.2 ng/mL) and overnight GH sampling. The MRI of the pituitary showed bilateral low grade OPGs, with an unremarkable pituitary gland and hypothalamus.

#### 3.1.4. Patient 4

A 3-year-old Caucasian female with NF1 was presented for the evaluation of accelerated linear growth. Her growth velocity was 11.2 cm/year. She had no evidence of precocious puberty on physical examination. Biochemical screening revealed IGF-1 of 353 ng/mL (Z-Score for Tanner 1: 6.5), and GH 3.5 ng/mL. GH excess was confirmed by failure to suppress GH on OGTT (GH nadir = 2). Brain MRI showed gliomas in the optic tracts, optic nerve, and optic chiasm, and a hypothalamic mass extending into third ventricle and bilateral basal ganglia. A partial resection of the glioma was performed, which revealed a low-grade pathology.

#### 3.1.5. Patient 5

**Figure 2:**
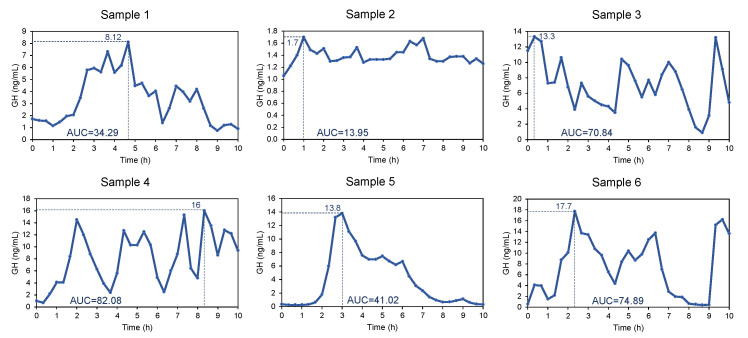
Overnight sampling of GH in six patients with NF1 and GH excess. An exaggerated pattern of blunted peaks in patient 2 (blue line) was observed. Red line = mean.

A 52-year-old Caucasian male with NF1 diagnosed at the age of 36 after workup of a left adrenal pheochromocytoma and a non-functional pNET was presented for the evaluation of acromegaly. His clinical features included the progressive enlargement of hands, feet, lips, testes, and neurofibromas. Biochemical screening revealed IGF-1 of 538 ng/mL (87–238, Z-score: 9.9), and GH 2.32 ng/mL. GH excess was confirmed by failure to suppress GH on OGTT (GH nadir = 1.45 ng/mL) and overnight GH sampling. We observed an exaggerated pattern of serial GH, which consisted of blunted peaks with mean GH levels of 1.4 ± 0.14 ng/mL throughout sampling (Figure 2, sample 2), when compared to the normal pattern, which consists of intermittent bursts of GH secretion during the night and a part of the day, with concentrations varying from undetectable to very high values [36]. The MRI of the brain showed spongiform gliosis or low-grade gliomas involving the right basal ganglia and thalami, with a voluminous pituitary gland and an unremarkable hypothalamus.

#### 3.1.6. Patient 6

A 3-year-old Caucasian male with NF1 was presented for the evaluation of accelerated linear growth. His growth velocity was ~8 cm/year. Testes were enlarged at 5cc and his LH was pubertal at 1.4 mIU/mL; leuprolide acetate was initiated for central precocious puberty. Biochemical evaluation following this therapy showed IGF-1 of 683 ng/mL (Z-Score for Tanner 1: 15.9), and GH 9.9 ng/mL. GH excess was confirmed by failure to suppress GH on OGTT (GH nadir = 8.8 ng/mL). Pituitary MRI revealed bilateral OPGs, a hypothalamic lesion likely representing a glioma, and an unremarkable pituitary gland and hypothalamus.

#### 3.1.7. Patient 7

A 29-year-old Caucasian female with NF1 first diagnosed at age 3 was presented for the evaluation of secondary amenorrhea. Her menses were irregular since age 12; she experienced complete amenorrhea in her early 20s. She had been noting progressive enlargement in hands and feet (shoe size 12) with an inability to fit her old rings into her fingers. She had the NF1 microdeletion syndrome, characterized by a more severe phenotype, and presenting with variable facial dysmorphism, and clinical features, including a large number of neurofibromas for her age [37]. Biochemical evaluation revealed IGF-1 of 451 ng/mL (117–329, Z-score: 4.3), and GH 4 ng/mL. Overnight GH and prolactin sampling confirmed elevations in both (Figure 2, sample 5; prolactin graph shown in Figure S1, sample 7). Pituitary MRI showed a Rathke’s cyst with a voluminous pituitary gland, suggestive of somatotroph hyperplasia, the first report of its kind in a patient with NF1 and GH excess [13].

#### 3.1.8. Patient 8

A 14-year-old Hispanic male was presented for the evaluation of delayed puberty. He had noted progressive fatigue, somnolence, headaches, decreased visual acuity, and his dentist had noted prognathism. Biochemical evaluation revealed IGF-1 of 268 ng/mL (Z-Score for Tanner 3: −0.62), and GH 6 ng/mL. Pituitary MRI revealed a macroadenoma (3.3 × 2.8 × 2 cm) with suprasellar extension, displacing the optic chiasm and extending along the cavernous sinus bilaterally. GH excess was confirmed by failure to suppress GH on OGTT (GH nadir = 4.5 ng/mL). He had a partial transsphenoidal resection of the tumor and on histopathology the immunostaining was positive for GH and prolactin. The pathological diagnostic was a large invasive somatolactotroph tumor.

#### 3.1.9. Patient 9

A 42-year-old female with multiple skin neurofibromas, Lisch nodules, and multinodular goiter was evaluated for acromegaly given her acral growth and coarse facial features. Biochemical evaluation revealed IGF-1 of 958 ng/mL (95–390, Z-score: 9.7), and GH of 3.1 ng/mL. GH excess was confirmed by failure to suppress GH on OGTT (GH nadir = 3). Pituitary MRI revealed a macroadenoma with size 1.4 × 1 cm. She was reluctant to surgery and opted for medical treatment.

#### 3.1.10. Patient 10

A 5-year-old girl with NF1 and OPG was evaluated for GH excess given a growth acceleration of 11 cm/year. She had no evidence of precocious puberty on physical examination. Optic nerve examination indicated early optic neuropathy. Biochemical evaluation revealed IGF-1 of 270 ng/mL (Z score for Tanner 1 = 3.5). GH excess was confirmed by failure to suppress GH on OGTT (GH nadir = 11.6 ng/mL), and overnight GH sampling. Pituitary and brain MRI showed bilateral OPGs and multifocal supra- and infra-tentorial myelin vacuolization.

### 3.2. Biochemical Testing

All patients met the biochemical criteria for the diagnosis of GH excess. The serial overnight sampling of GH levels in six patients revealed a mean of 4.5 GH peaks, an elevated nadir GH level of 0.68 ± 0.27 ng/mL, a mean GH level of 5.2 ± 2.3 ng/mL, with a mean AUC between 8 PM and 6 AM of 54.7 ± 24.2 ng/mL·h (Figure 2 and Table 3).

### 3.3. Genetic Results

Germline alterations in the *NF1* gene were identified in nine patients, as listed in Table 4. Patient 1 has no genetic analysis available, but met the NF1 diagnostic criteria, as detailed elsewhere [30]. The other patients harbored either a heterozygous pathogenic single nucleotide variant (SNV) in *NF1* or a *NF1* deletion.

Three patients (#3, 4, and 10) had a truncating SNV. It is worth noting that the variant we report in patient 3 (c.6406dupT, p.Ser2136Phefs*12) is described here for the first time.

Four patients (#5, 6, 7, and 9) had a missense SNV. Patients 5 and 9 harbored the same variant (c.2329T>A, p.Trp777Arg), predicted by in silico analysis to be likely damaging to the protein/structure function. Interestingly, another nucleotide substitution (c.2329T>C) resulting in the same amino acid change has also been reported in a Chinese Han family with NF1 [38]. The *NF1* variant we found in patient 8 (c.1541A>C, p.Gln514Pro) leads to a non-conservative amino acid substitution (Grantham dist.: 76, reference range of 0–215) and occurs in a position evolutionary conserved in mammals. In silico analysis is inconsistent in its predictions; therefore, this variant should be considered of uncertain significance (VUS).

Patient 2 had a novel heterozygous *NF1* deletion (c.(576_617) (785_958)del), while patient 7 had a 1.3 Mb deletion at 17q11.2 affecting 21 genes and associated with a contiguous gene deletion syndrome, which is found in a range from 5 to 20% of patients with *NF1* (NF1 microdeletion syndrome, MIM: 613675).

We were only able to perform LOH studies for *NF1* in one pituitary tumor (Patient 8, Table 3), given poor quality or absence of DNA from other samples and/or patients. No LOH for *NF1* was observed in patient 8′s pituitary tumor DNA.

### 3.4. Immunohistochemical Testing

**Figure 3:**
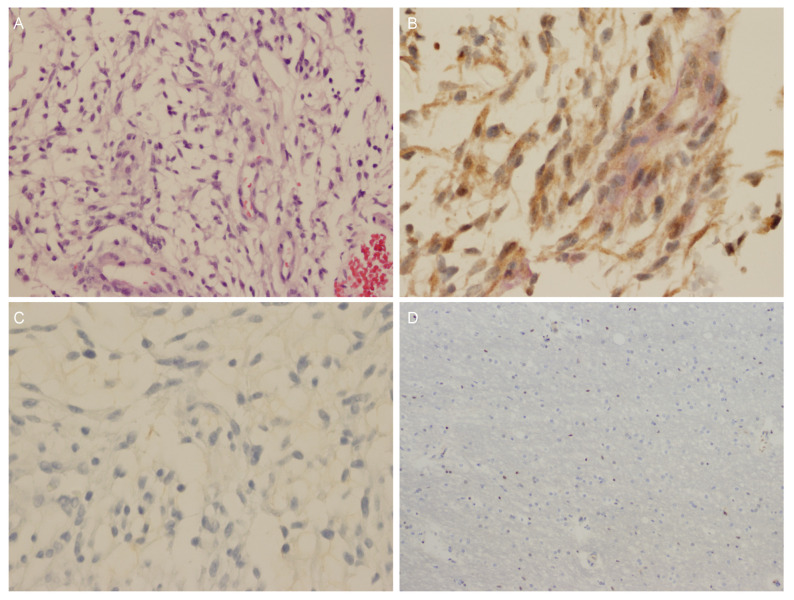
Partially resected optic pathway glioma (OPG) from patient 4 demonstrating an overexpression of GPR101. (**A**) H&E staining (20×): low grade OPG. (**B**) GPR101 staining (brown, 40×): strong cytoplasmic signal. (**C**) GH staining (40×): negative. (**D**) Control optic nerve sample with negative staining for GPR101 (10×).

Six patients had OPGs that were detected on imaging, three patients had a pituitary tumor (two available for analyses), and one pediatric patient had both a pituitary tumor as well as an OPG. Patient 4 had an OPG that stained strongly for GPR101 (Figure 3) and weakly for the GH receptor (GHR), but did not stain for GHRH, GH or somatostatin (previously published [12]). This OPG showed an expression of GPR101 that is stronger than five optic nerve controls (5 micron sections on charged slides from autopsy collected during brain autopsies for other diseases; a representative image is shown in Figure 3D).

**Figure 4:**
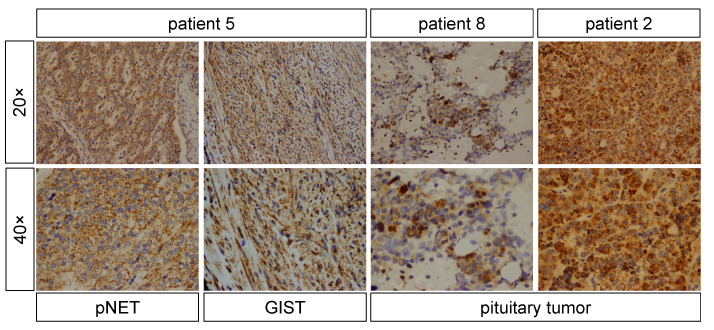
Immunohistochemical staining for NF1 showed positivity in the pNET and GIST of patient 5, and in the pituitary tumors of patients 8 and 2.

The non-functional pNET and GIST from patient 5 showed positive staining for NF1 in pNET and GIST (Figure 4), and negative GH staining in the pNET (data not shown). This patient had a non-infiltrating OPG and a voluminous pituitary gland, suggestive of hyperplasia. The pituitary tumor from patient 8 stained positive for reticulin, GH, prolactin (data not shown), and NF1 (Figure 4). The pituitary tumor from patient 2 stained positive for NF1 (Figure 4) and negative for GH (data not shown). Patient 9 opted for medical therapy. Patients 5 and 7 had a voluminous pituitary gland, highly suggestive of somatotroph hyperplasia as the likely mechanism of GH excess.

## 4. Discussion

We examined ten patients with NF1 and GH excess, including six children who had OPGs (one with a concomitant pituitary tumor), one adolescent and one adult with a pituitary tumor without an OPG, and two adults with suggestive pituitary hyperplasia (one with a concomitant OPG). The serial overnight sampling of the GH levels in six patients confirmed GH excess with pattern variability; interestingly, an exaggerated pattern of GH secretion was observed in patient 5, which consisted of blunted peaks, with mean GH levels of 1.4 ± 0.14 ng/mL. Elevations in both GH and prolactin in patient 7, who, as in the case of patient 5, also had a voluminous pituitary gland, was suggestive of somato-lactotroph hyperplasia (secretion of GH and prolactin likely by two different cell lines rather than somatomammotroph hyperplasia). These important findings are the first to support the potentially causal association and wide clinical, biochemical, and pathological spectrum of pituitary tumorigenesis leading to GH excess in NF1.

GH excess, leading to gigantism or acromegaly, is usually caused by a somatotroph tumor (or less commonly somatotroph hyperplasia) in most cases [39]. The remaining causes are due to the occurrence of hypothalamic lesions [5,23], or non-pituitary tumors (i.e., NETs) secreting GH or GHRH [40].

GH excess has been reported in children and adults with NF1, although the exact incidence or mechanism is unknown [5,6,7,8,9,10,11,12,13,14,15,16]. It has been suggested that the loss of somatostatinergic inhibition from infiltrating OPGs into somatostatinergic pathways leading to dysregulated GH secretion is the most plausible mechanism leading to overgrowth [5,22]. It is believed that normal projections of somatostatinergic areas of the hypothalamus exert an inhibitory influence on GH release [41], and are presumed to be lost with infiltrating OPGs. This was further supported by a study that showed negative OPG staining for GHRH, GH, and somatostatin and weakly positive staining for GH receptors [12]. However, this mechanism may not fully explain all the cases of NF1 with GH excess that have been described to date [7,8,9,10,13,18,19,20], particularly in those without an infiltrating OPG. In our cohort, based on imaging studies, most OPGs did not seem to be of the infiltrating type, suggesting other potential mechanisms for GH excess. Moreover, the hypothesis of a co-occurring somatotroph tumor or hyperplasia has not been genetically ascertained in NF1 with GH excess [15,16].

Pituitary tumors in patients with NF1 have been previously described [8,9,10,13,18,19,20,42,43,44,45]. However, the co-existence of a somatotroph tumor or hyperplasia in patients with NF1, with or without a concomitant OPG, has not been recognized previously. In two previous case reports of patients with NF1 and GH excess, a pituitary tumor was reported as a serendipitous association [8,10]. Interestingly, the described cases of co-existing NF1, GH excess, and OPG to date had normal appearing pituitary glands on radiography [6,16], without evidence of tumor or hyperplasia.

NF1 is a disorder that predisposes to neoplasia in tissues derived from embryonic neural crest. Recently, a small proportion of hormone-secreting pituitary cells were found to originate from the neural crest [46]. Thus, it could be speculated that genetic defects in *NF1* have the potential to cause pituitary lesions.

In our cohort, out of seven patients (60%) with OPGs, only three (# 2, 4 and 6) had hypothalamic infiltration. Indeed, patient 2 had optic and hypothalamic lesions, likely representing gliomas, and a 4 mm right pituitary tumor that was resected with pathology negative for GH and biochemically persistent GH excess post-surgery, likely due to increased hypothalamic GHRH release versus the loss of somatostatin tone from the infiltrating lesions.

The lack of LOH for *NF1* in the only pituitary tumor (patient 8) that we could test seems to argue against our hypothesis of a causal association between NF1 and pituitary tumorigenesis. However, we could not exclude that *NF1* was lost in the tumor via a compound heterozygous mutation or microdeletion since the fragmented tumor DNA at our disposal did not allow these types of investigations. Moreover, that was the only patient harboring a *NF1* variant of uncertain significance. The lack of available DNA from the other two pituitary tumors precluded us from drawing definitive conclusions.

Pituitary hyperplasia leading to GH excess was long thought to be due to a GHRH-secreting NET [47]. However, growing evidence has demonstrated that several syndromic conditions, including the McCune–Albright syndrome (MAS) [48,49], Carney complex (CNC) [50,51], and rarely multiple endocrine neoplasia type 1 (MEN1) [52] and familial isolated pituitary adenomas (FIPA) [53], lead to GH excess through pituitary hyperplasia that appears to precede tumor development. Thus, in at least some patients with NF1 and GH excess, the pituitary gland could be characterized by somatotroph tumor or hyperplasia. Our data underline the need for the early recognition and investigation for gigantism or acromegaly in patients with NF1, including a careful and thorough investigation of the pituitary gland through imaging using techniques for three dimensional volumetric measurements [54], and serial overnight GH and prolactin measurements, if feasible. The presence of a pituitary tumor or hyperplasia in NF1 with GH excess may indeed alter the treatment and follow-up of these patients.

The most frequent neoplasm in NF1 during the first six years of life is an OPG [55], affecting <20% of patients [17]. Usually, OPGs are asymptomatic, do not require intervention, and do not show clinical progression. However, low-grade OPGs affecting the hypothalamus and suprasellar areas may evolve over decades and present with GH deficiency (40.3%), central precocious puberty (26.0%), gonadotropin (20.4%), TSH (13.3%), and ACTH (13.3%) deficiency [56,57,58], or GH excess (10%) [14]. These comorbidities may also be observed in children or adults without MRI evidence of OPGs [59,60]. A retrospective study of 36 children with NF1 and OPGs showed that 2/36 (5.6%) of the cohort had GH excess [16]. In another study, three children with NF1, GH excess and OPGs involving the hypothalamus and optic chiasm were reported [12]. The same investigators later showed that some patients had a resolution of GH excess without treatment, suggesting a transient or fluctuating course of GH excess in this at-risk population [24]. Reversibility was also confirmed by a subsequent report of two cases of NF1 with GH excess and OPGs [6]. Large and clinically symptomatic OPGs in NF1 may undergo spontaneous regression with a variable degree of improvement in the visual function [61]. However, recent estimates suggest that up to 10% of patients with NF1 and OPG have GH excess [14]. Interestingly, in our cohort, out of seven patients with an OPG, six had gigantism. The age at NF1 diagnosis either coincided or shortly preceded that of GH excess, suggesting a common pathogenetic mechanism.

Our group and others have recently shown that patients harboring *GPR101* microduplications present with early childhood-onset gigantism (X-LAG) [26,62]. GPR101 is a GPCR important for brain and pituitary development [28] that was shown to be highly expressed in the GH- and prolactin-secreting pituitary lesions of X-LAG patients [26,63]. We investigated the potential role of GPR101 from the partially resected OPG of patient 4, a 3-year-old Caucasian female with NF1. This patient showed a growth velocity of 11.2 cm/year in the context of several gliomas in the optic tracts, nerve, chiasm and radiation, and a hypothalamic mass extending into third ventricle and bilateral basal ganglia, and was previously published [12]. Immunohistochemical staining for GPR101 in the OPG showed strong staining with a cytoplasmic signal when compared to no-to-very-low staining in control optic nerves. Supporting our finding, GPR101 mRNA is normally not expressed in NF1-related OPGs not associated with GH excess [64]. In rodents, *Gpr101* is highly expressed in the hypothalamus, and may play a role in the hypothalamic control of energy homeostasis, and physiological processes associated with pregnancy, lactation, and reproduction [26,65,66,67]. The higher expression levels observed in patient 4’s OPG not harboring a duplication of *GPR101* compared to normal optic tract tissues suggest that *GPR101* over-expression is likely caused by other yet-to-be identified genetic or epigenetic mechanisms and that GPR101 might be implicated in the growth of the OPG and perhaps GH excess. A study by Daly et al. [68] showed that the pathology of X-LAG includes the hypothalamic dysregulation of GHRH secretion as a possible mechanism contributing to GH excess. We speculate that, in this patient, GPR101 expression in the OPG might stimulate the secretion of GHRH from nearby interacting neurons in the hypothalamic arcuate nucleus, leading to dysregulated GH secretion from the pituitary gland. However, GPR101 staining in patients with NF1 and OPG without GH excess has not been performed, and therefore establishing causality between GPR101 and NF1 remains to be proven in future studies. We were not able to find OPGs in individuals with and without NF1 for staining as OPGs are rarely resected or biopsied. Hence, at present, we cannot confirm our preliminary finding in additional tumors.

Clinicians caring for patients with NF1 should have a high index of suspicion for GH excess given the increased comorbidities associated with a delayed diagnosis [25]. Usually, children with clinical features of NF1, particularly café-au-lait spots, which develop in infancy in >95% of individuals [69], and a positive family history, can be identified within the first year of life. However, since many of the features of NF1 increase in frequency with age, and most children meet the diagnostic criteria for NF1 by age eight [70], the diagnosis of NF1 could be missed, particularly in sporadic cases affecting younger children [71]. In this study, five patients were diagnosed with GH excess before the age of 8 years, with a delay of 2.6 ± 1.8 years from the initial diagnosis of NF1. Thus, the diagnosis of GH excess in NF1 should be suspected in children with accelerated linear growth, clinical features of gigantism, such as enlargement of the hands and feet, soft-tissue thickening, prognathism, coarse facial features, and presence of an OPG, or worsening of clinical features, such as neurofibromas, pain or endocrinopathies in the context of NF1 [25]. Adults with GH excess and NF1 may also present with progressive clinical or coarse features suggestive of acromegaly, or worsening neurofibromas, pain or endocrinopathies [25]. It is important to note that short adult height is an important characteristic of NF1 [72]. Thus, clinicians should not be deterred from evaluating short patients with NF1 for GH excess. Conversely, patients with NF1 carrying large heterozygous deletions involving *NF1* and contiguous genes lying in its flanking regions are tall [73], although some patients with NF1 and tall stature (>90th percentile) could have a central nervous system tumor [72]. In children and adults with NF1, GH excess is confirmed with high levels of IGF-1, IGF-binding protein 3, and lack of GH suppression to levels <1 ng/mL, during OGTT [25]. Serial overnight GH (and prolactin) sampling—albeit difficult to perform since requiring specialized centers—is helpful in the workup of GH excess, particularly in cases where the initial testing of GH excess is not informative. Thus, minimizing future endocrine morbidity through early recognition and diagnosis remains an important aspect of managing patients with NF1.

A limitation of this study is that our retrospective case series is not representative of the whole population of patients with NF1, with or without and OPG. Hence, we cannot estimate the prevalence or incidence of GH excess in NF1 patients or confirm previous estimates [14,16].

## 5. Conclusions

We performed in this paper a comprehensive evaluation of pediatric and adult patients with NF1 and GH excess. Although the pathophysiology of GH excess in NF1 remains to be elucidated, we presented novel insights into potential pathogenetic mechanisms. Our study suggests that pituitary lesions, including somatotroph and/or somato-lactrotroph tumor or hyperplasia, might be an under recognized cause of GH excess in NF1. Additionally, GPR101, an orphan GPCR important for brain and pituitary development and implicated in X-LAG, was overexpressed in one OPG. Further studies are required to ascertain the functional link between NF1, GH excess and GPR101, in patients with this condition.

## Figures and Tables

**Table 1:** Clinical and biochemical characteristics of 10 patients with NF1 and GH excess.

ID	Sex	Age (Years) at NF1 Diagnosis	Age (Years) at GH Excess Diagnosis	Acromegaly	Height SDS	Random GH (ng/mL)	Random IGF-1 ^a^ (ng/mL)	GHRH ^b^ (pg/mL)
1	F	Birth	4.8	No	1.8	2.3	540 (Z-score: 9)	31
2	M	2	3	No	3	13.6	659 (Z-score: 15.3)	N/A
3	M	1	4	No	2.7	1.55	431 (Z-score: 3.8)	5
4	F	3	3	No	2.7	3.5	353 (Z-score: 6.5)	N/A
5	M	38	52	Yes	2.1	1.41	538 (Z-score: 9.9)	75
6	M	3	3	No	2.3	9.86	683 (Z-score: 15.9)	N/A
7	F	3	29	Yes	0.3	4.6	451 (Z-score: 4.3)	11
8	M	25	14	No	3.2	6	268 (Z-score: −0.62)	N/A
9	F	42	42	Yes	2.0	3.1	958 (Z-score: 9.7)	N/A
10	F	5 months	5	No	1.1	1.14	270 (Z-score: 3.5)	27

^a^ Calculated Z-Scores are listed by tanner stage 1, except for patient 8 (tanner stage 3) and adults. ^b^ GHRH reference values; GHRH secreting NET, 200–10,000 pg/mL; patients with acromegaly, up to 200 pg/mL. Test performed by Inter Science Institute, Inglewood, CA, USA. N/A, not available.

**Table 2:** Pathological characteristics of 10 patients with NF1 and GH excess.

ID	Pituitary MRI	Pathology	Family History of NF1
1	No tumor	N/A	Positive
2	Microadenoma (~5 mm)	Pituitary tumor stained negative for GH, patchy positive for GPR101, positive for NF1	Positive
3	No tumor	N/A	Negative
4	No tumor	OPG stained positive for GPR101 (cytoplasmic signal), negative for GHRH, GH, and somatostatin and weakly positive for GH	Positive
5	No tumor	GIST stained patchy positive for GPR101 and small nuclear staining in pNET	Positive
6	No tumor; hypothalamic infiltration	N/A	Negative
7	Voluminous pituitary gland with Rathke’s cyst	N/A	Negative
8	Macroadenoma (~1 × 0.5 cm)	Pituitary tumor stained positive for GH, PRL and NF1, and patchy positive for GPR101	Negative
9	Macroadenoma (~1.4 cm)	N/A	Negative
10	No tumor; Right inferior hypothalamic enhancement	N/A	Negative

Abbreviations: GH, growth hormone; GPR101, G protein-coupled receptor 101; N/A, not applicable; NET, neuroendocrine tumor, “p” for pancreatic; NF1, neurofibromatosis type 1; OPG, optic pathway glioma; PRL, prolactin; GIST, gastrointestinal stromal tumor.

**Table 3:** Overnight sampling of GH in six patients with NF1 and GH excess.

	Sample 1	Sample 2	Sample 3	Sample 4	Sample 5	Sample 6
Patient	3	5	2	1	7	10
N	31	31	31	31	31	31
Mean GH (ng/mL)	3.3	1.4	7.1	8.1	3.9	7.5
95% CI	2.6–4.1	1.3–1.4	5.913–8.3	6.4–9.7	2.5–5.5	5.5–9.6
Variance	4.2	0.01	10.8	19.6	17.0	29.3
SD	2.0	0.1	3.3	4.4	4.1	5.4
Minimum GH (ng/mL)	0.8	1.1	0.9	0.7	0.3	0.4
Maximum GH (ng/mL)	8.1	1.7	13.3	16	13.8	17.7
AUC (baseline = 0; ng/mL·h)	34.3	13.9	70.8	82.1	41.0	74.9

**Table 4:** Genetic characteristics of our NF1 population.

ID	*NF1*							In Silico Prediction	Other Genetic Findings	Ref.
	DNA Change	Protein Change	dbSNP ID	ClinVar ID	Location in Gene	LOH	Total MAF (%)			
1	N/A	N/A	N/A	N/A	N/A	N/A	N/A	N/A	N/A	[17]
2	c.(576_617)_(785_958)del	N/A	N/A	N/A	exons 6–9	N/A	N/A	pathogenic	No *GPR101* SNVs or CNVs	N/A
3	c.6406dupT	p.Ser2136Phefs*12	N/A	N/A	exon 42	N/A	N/A	pathogenic	N/A	N/A
4	c.6791dupA	p.Tyr2264*	rs876657715	RCV000213933.4 RCV000486063.1	exon 45	N/A	N/A	pathogenic	No *GPR101* SNVs or CNVs, 57 kb deletion at 6q12	[12]
5	c.2329T>A	p.Trp777Arg	rs876658853	RCV000219741.1	exon 20	N/A	N/A	likely pathogenic	N/A	[21]
6	c.2339C>G	p.Thr780Arg	rs199474746	VCV000457581	exon 20	N/A	N/A	pathogenic	N/A	[22]
7	17q11.2(29,039,980–30,352,755) × 1	N/A	N/A	N/A	entire gene	N/A	N/A	pathogenic	N/A	N/A
8	c.1541A>C	p.Gln514Pro	rs775369084	VCV000232968.4	exon 14	no	0.01349	VUS	No *GPR101* SNVs, no *AIP* SNVs or CNVs, no *MEN1* SNVs or CNVs	N/A
9	c.2329T>A	p.Trp777Arg	rs876658853	RCV000219741.1	exon 20	N/A	N/A	likely pathogenic	N/A	N/A
10	c.7285C>T	p.Arg2429*	rs786202457	RCV000414562.1 RCV000165277.1 RCV000414830.1 RCV000532076.6 RCV000999932.1	exon 49	N/A	N/A	pathogenic	N/A	[23]

LOH: loss of heterozygosity (in the pituitary tumor); MAF: minor allele frequency (from gnomAD); SNVs: single nucleotide variants; CNVs: copy number variants; N/A: not available/applicable; VUS: variant of uncertain significance.

## Data Availability

Not applicable.

## Supplementary Materials

The following supporting information can be downloaded at: https://www.mdpi.com/article/10.3390/jcm11082168/s1. Figure S1: Overnight sampling of prolactin in two patients (#3 and 7) with NF1 and GH excess. Elevated prolactin levels in patient 7 (blue line) were observed.
