# Supplementary material for: Neurofibromatosis Type 1 Has a Wide Spectrum of Growth Hormone Excess
Source: J Clin Med. 2022 Apr 13;11(8):2168. doi: 10.3390/jcm11082168 (PMC9029762; doi:10.3390/jcm11082168)
Supplement: Supplementary file 1 [file jcm-11-02168-s001.zip › jcm-1625681-supplementary.pdf]

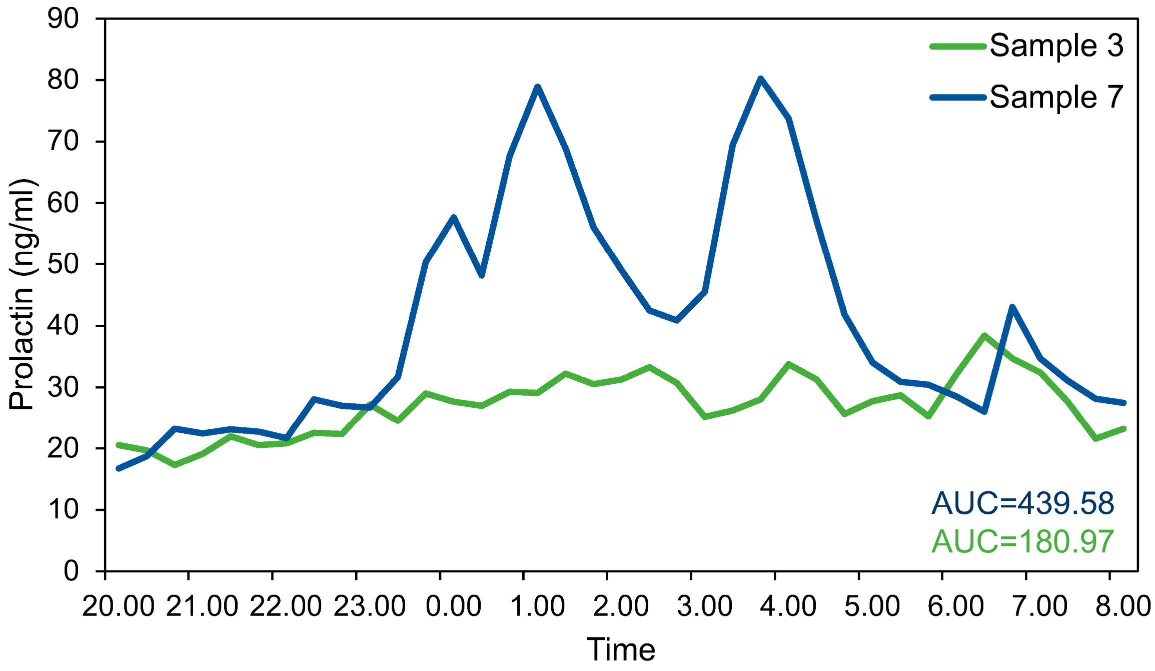

Figure S1. Overnight sampling of prolactin in two patients (#3 and 7) with NF1 and GH excess. Elevated prolactin levels in patient 7 (blue line) were observed.
